# SRSF3 Promotes Angiogenesis in Colorectal Cancer by Splicing SRF

**DOI:** 10.3389/fonc.2022.810610

**Published:** 2022-02-07

**Authors:** Yinshuang Chen, Man Yang, Fanyi Meng, Yawen Zhang, Mengmeng Wang, Xuqin Guo, Jie Yang, Hongjian Zhang, Haiyang Zhang, Jing Sun, Weipeng Wang

**Affiliations:** ^1^Center for Drug Metabolism and Pharmacokinetics, College of Pharmaceutical Sciences, Soochow University, Suzhou, China; ^2^Institute of Medical Technology, Suzhou Vocational Health College, Suzhou, China

**Keywords:** angiogenesis, colorectal cancer, splicing, SRF, SRSF3

## Abstract

SRSF3, an important member of the serine/arginine-rich protein (SRp) family, is highly expressed in various tumors and plays an important role in tumor cell proliferation, migration and invasion. However, it is still unclear whether SRSF3 is involved in tumor angiogenesis. In this study, we first revealed that SRSF3 regulated the expression of numerous genes related to angiogenesis, including proangiogenic SRF. Then, we confirmed that SRSF3 was highly expressed in colorectal cancer (CRC) and was positively correlated with SRF. Mechanistic studies revealed that SRSF3 directly bound to the “CAUC” motif in exon 6 of *SRF* and induced the exclusion of introns. Knockdown of SRSF3 significantly reduced the secretion of VEGF from CRC cells. Conditioned medium from SRSF3-knockdown CRC cells significantly inhibited the migration, invasion and tube formation of human umbilical vein endothelial cells (HUVECs). In addition, SRF silencing inhibited angiogenesis, while SRF overexpression reversed the antiangiogenic effects of SRSF3 knockdown on tube formation. These findings indicate that SRSF3 is involved in the splicing of *SRF* and thereby regulates the angiogenesis of CRC, which offers novel insight into antiangiogenic therapy in CRC.

## Introduction

CRC is the third most common cancer in the world, and its mortality rate ranks the second (1). Approximately 86% of advanced CRC patients still relapse within 5 years after surgery, and most of these recurrences are in the form of metastasis (2, 3). Moreover, metastatic CRC is difficult to overcome (4). The blood vessels in tumors can transport oxygen and nutrients to promote tumor growth, so angiogenesis is one of the hallmarks of cancer (5). Moreover, angiogenesis plays a vital role in CRC development and has been used as a potential target for metastatic CRC treatment (6, 7). Recently, antiangiogenic therapy has made a major breakthrough in metastatic CRC treatment, significantly prolonging the survival time of patients (4, 8, 9). For example, monoclonal antibodies cetuximab and panitumumab that block epidermal growth factor (EGFR) (10), bevacizumab, aflibercept, ramoximab and regofenib that block vascular endothelial growth factor (VEGF) and VEGF receptor (11–13) have achieved great therapeutic effects in the clinical treatment of patients with metastatic CRC (14). However, there are still many problems in the antiangiogenic therapy of CRC, such as limited scope of application, drug resistance and poor prognosis (11, 15).

Alternative splicing is a critical mechanism to generate diverse structural and functional proteins (16), and more than 95% of human genes undergo alternative splicing. Alternative splicing occurs in various biological processes related to cancer, including invasion, metastasis and angiogenesis (17). Therefore, aberrant splicing could be used not only as a marker of cancer but also as a potential target for cancer treatment (18). Oncogenic mRNA transcripts are produced by the interaction of splicing factors and pre-mRNA. For example, the serine/arginine-rich (SR) proteins SRSF1 and SRSF5 promote the splicing of vascular endothelial growth factor A (*VEGFA*) and generate proangiogenic isoforms, which are upregulated in tumors (19). Splicing factor 3a subunit 3 (SF3A3) accelerates the production of platelet-derived growth factor receptor (*PDGFR*), which is associated with angiogenesis (20). RNA binding motif protein 4 (RBM4) and SRSF1 motivate the production of hypoxia inducible factor 1 subunit alpha (*HIF-1*α) full length isoform and *Δ14 HIF-1α* isoform, which are proangiogenic (21). ESRP1 enhances the expression of mesenchymal spliced variant *CD44s* (standard), which plays an important role in the epithelial-mesenchymal transition (EMT) process of epithelial ovarian cancer (22).

Splicing factor SRSF3, an important member of the serine/arginine-rich protein (SRps) family, contains an N-terminal RNA-binding domain and a downstream SR-rich domain. It binds to RNA and acts as a regulator of alternative splicing for many genes. SRSF3 has been identified as a proto-oncogene and overexpressed in multiple cancers (23–25). For example, SRSF3 acts as a *PKM* splicer and plays a positive role in cancer-specific energy metabolism in CRC (26). SRSF3 also affects the expression of spliced variant coiled-coil domain containing 50 short (*CCDC50S*) to contribute to the growth and metastasis of hepatocellular carcinoma (HCC) through the Ras/Foxo4 signaling pathway (27). Recent studies have suggested that SRSF3 is a significant regulator of glioblastoma-related alternative splicing and is directly associated with glioblastoma development, progression, aggressiveness and patient survival. It represents a novel potential therapeutic target to tackle this devastating pathology (28, 29). However, the role of SRSF3 in CRC angiogenesis is still unclear.

In this study, we demonstrated that SRSF3 regulated the expression of numerous genes related to angiogenesis. We subsequently identified the proangiogenic role of SRSF3 in CRC. Furthermore, we showed that SRSF3 regulated serum response factor (SRF) expression by binding to the “CAUC” motif in exon 6 of *SRF* pre-mRNA and participated in the splicing of *SRF*. Moreover, we confirmed that SRF had a proangiogenic effect and that SRSF3 promoted the angiogenesis of CRC by splicing *SRF*.

## Methods

### Cell Culture

HCT-116 and HCT-8 cell lines (ATCC, USA) were cultured in DMEM (HyClone, USA) with 10% fetal bovine serum (FBS, Gibco). Human umbilical vein endothelial cells (HUVECs) were a gift from the Institute for Cardiovascular Science of Soochow University and cultured in DMEM/F12 (HyClone, USA) containing 10% FBS. All cells were cultivated at 37°C in a humidified incubator containing 5% CO_2_.

### siRNAs and Plasmids

*SRSF3*-specific siRNA, *SRF*-specific siRNA and siRNA control were purchased from GenePharma. The human Flag-tagged *SRSF3* expression plasmid was cloned into the pcDNA3.1 (+) vector and synthesized by GENEWIZ. The human Flag-tagged *SRF* expression plasmid was cloned into the GV492 vector, which was synthesized by GeneChem. Minigene recombinant plasmids expressing *SRF* exons 5-7 with or without point mutations were synthesized by Synbio Technologies. siRNAs and plasmids were transfected into HCT-116 and HCT-8 cell lines using Lipofectamine 2000 (Invitrogen, USA) according to the manufacturer’s protocol.

### RT–PCR and qRT–PCR

Total RNA was isolated from CRC cells using RNAiso Plus (#9109, Takara), and the quantity was measured with a NanoDrop 2000 spectrophotometer (Thermo). cDNA synthesis was performed from 1000 ng of total RNA with an RT-Kit (Thermo) and random primers (TaKaRa) as described by the manufacturer. An equal amount of cDNA was amplified by PCR using premix Taq™ (TaKaRa) and separated on an agarose gel. Signal intensities of ethidium bromide-stained bands were quantified using ImageJ software. Specific mRNA expression was measured by qPCR using SYBR Green (Bio–Rad) operated on the Bio–Rad CFX96-C1000, and the relative RNA amount was calculated by the 2^-ΔΔCt^ method with normalization to *GAPDH*. Primer sequences are shown in **Supplementary Table 1**.

### Western Blotting

Protein samples were extracted from the cells with RIPA buffer (Beyotime) containing a protease and phosphatase inhibitor mixture. The extracted proteins were separated by 10% sodium dodecyl sulfate–polyacrylamide gel electrophoresis and then transferred onto 0.45 μm PVDF membranes (Merck Millipore). After blocking for 1.5 h at room temperature with 5% nonfat milk, the membranes were incubated overnight at 4°C with primary antibodies against SRSF3 (ab198291, Abcam, 1:10000), SRF (16821-1-AP, Proteintech, 1:1000), GAPDH (AF0006, Beyotime, 1:1000) and β-actin (AF0003, Beyotime, 1:1000). Following incubation with an appropriate HRP-conjugated secondary antibody for 1 h at room temperature, the proteins on the membranes were detected with an ECL Western Blotting Detection System (Merck Millipore). The intensities of bound antibodies were quantified using ImageJ software.

### RNA Sequencing

HCT-116 cells were transfected with *SRSF3* siRNA and siRNA control for 48 h, and total RNA was extracted as described above. Then, library construction and RNA sequencing (RNA-seq) were performed by Shanghai OE Biotech, followed by computational analysis. Briefly, the count number of each gene was normalized and then the fold change (*F*c) was calculated by using DESeq software. The significance of the difference in the number of reads was tested by using negative binomial distribution test. Genes were identified as significantly differentially expressed with nominal *P*-value <0.05 and *F*c > 2 or < 0.5.

### CRC Tissue Samples

Fifty-five CRC tissue samples were collected by the Second People’s Hospital of Changshu from March 2018 to February 2019. All samples were stained with hematoxylin-eosin and confirmed by two pathologists. None of the patients had received chemotherapy or radiotherapy before surgery. The Ethics Committee of Soochow University approved all aspects of this study, and all patients signed informed consent forms.

### Immunohistochemistry

Immunohistochemistry (IHC) staining was performed by Servicebio (Wuhan) on human CRC tissues. Briefly, CRC tissues were cut into 4 µL-thick sections, deparaffinized in xylene, hydrated in ethyl alcohol and washed in tap water in an orderly manner. Next, the sections were incubated with SRSF3 and SRF antibodies (Santa Cruz). Finally, the sections were visualized under an inverted microscope (Olympus, Japan). The intensity of staining was reviewed by two independent pathologists. For each section, the stain strength was scored at 0-4, and the staining extent was scored as follows: 0 = 0% of tumor cells were positive staining, 1 = 1%-25% of cells were positive staining, 2 = 26%-50% of cells were positive staining, 3 = 51%-75% of cells were positive staining, or 4 = 76%-100% of cells were positive staining. The expression levels of SRSF3 and SRF were classified as negative (score 0), low (score 1-2), and high (score 3-4), respectively.

### Conditioned Medium

HCT-116 or HCT-8 cells were seeded in a 6-well plate (3.5×10^5^ cells/well for transfection with siRNAs or 7×10^5^ cells/well for transfection with plasmids). At 48 h after transfection, the medium in the plate was replaced with fresh medium containing 1% FBS and incubated for 24 h. Then, the conditioned medium (CM) was collected and centrifuged at 800 rpm for 10 min to remove cells and cell debris. The supernatant was stored at -80°C for subsequent ELISAs and HUVEC proliferation, migration, invasion and tube formation assays.

### ELISA

The concentration of VEGF in cell culture medium from HCT-116 or HCT-8 cells was analyzed by using a human VEGF ELISA kit (70-EK183-96, LiankeBio) according to the manufacturer’s instructions. Briefly, 1× washing solution was added to a 96-well microtiter plate at 300 μL per well and soaked for 30 s. Then, 100 μL standards and samples were successively added to the plate after discarding the washing solution. At the same time, 50 μL antibody was added to each well, the microtiter plate was sealed with a sealing membrane, and then the plate was placed in a constant temperature shaker at 25°C for 2 h. Coated wells were washed 6 times with 300 μL washing solution and blocked with 100 μL horseradish peroxidase-labeled streptavidin at 25°C for 45 min. After washing 6 times, 100 μL chromogenic substrate was added to each well and incubated for 5-30 min in a dark place at room temperature. Finally, 100 μL stop solution was added to each well. The OD values of the samples in the plate were measured at a maximum absorption wavelength of 450 nm and a reference wavelength of 570 nm or 630 nm by using an enzyme plate analyzer.

### Cell Proliferation Assay

Approximately 5000 HUVECs were seeded into a 96-well plate and incubated with the collected CM in a 37°C incubator with 5% CO_2_ for 24 h. Then, 10 μL MTT solution (5 mg/mL) was added to each well, and the cells were further cultured in the incubator for 2 h. After that, the medium containing MTT was removed, and 150 μL DMSO was added to each well. Finally, the 96-well plate was placed on a constant temperature shaker (800 rpm) at 37°C for 10 min, and the OD value at 492 nm was measured.

### Cell Migration and Invasion Assay

For the migration assay, 3×10^4^ HUVECs in 200 μL serum-free medium were seeded into the upper chamber (8 μm pore size, 24-well, #3422, Corning). Then, 600 μL CM was added to the lower chamber and incubated for 24 h at 37°C with 5% CO_2_. To assess the invasive capacity of HUVECs, the upper chambers were first coated with 50 μL diluted Matrigel (200 μg/mL, #356234, Corning). Then, 5×10^4^ HUVECs in 200 μL serum-free medium were seeded into the upper chamber, 600 μL CM was added to the lower chamber and incubated in a 37°C incubator with 5% CO_2_ for 48 h.

After 24 h of incubation for the migration assay or 48 h for the invasion assay, cells that could not migrate and invade were removed with a cotton swab from the upper part of the Transwell, and the inserts were fixed with 4% paraformaldehyde for 30 min at room temperature. Transwell inserts were stained in 600 μL 0.1% crystal violet solution for 20 min, and the stained cells were counted in at least three randomly selected fields at 100× magnification under a microscope to minimize bias.

### HUVEC Tube Formation Assay

A 96-well plate was coated with 50 μL Matrigel and incubated for 1 h at 37°C. Then, 2×10^4^ HUVECs in 100 μL CM were seeded into 96-well plates. After the plate was incubated for 10 h at 37°C, the tube structures were photographed using an inverted microscope and analyzed by Image-Pro Plus software. Three independent experiments were required for each treatment.

### RNA Immunoprecipitation Assay

Protein-A/G beads were first preincubated with 5% BSA of NT2 buffer (50 mM Tris-HCl, 150 mM NaCl, 1 mM MgCl_2_ and 0.05% NP-40, pH 7.4). Then, 3 μg SRSF3 antibody or control rabbit IgG (#A7016, Beyotime) was added and incubated at 4°C overnight to obtain SRSF3 antibody or rabbit IgG grafted beads. The beads were collected by centrifugation at 4°C and washed with NT2 buffer 5 times. HCT-116 cells cultured in 100 mm petri dishes were washed twice with 5 mL ice-cold PBS and then lysed in lysis buffer containing 100 mM KCl, 5 mM MgCl_2_, 10 mM HEPES (pH 7.0), 0.5% NP-40, 1 mM DTT, 1 mM PMSF and 100 U/mL RNase inhibitor for 30 min. After that, the lysis solution was collected by centrifugation. Subsequently, the cell lysate and the antibody-conjugated beads were incubated at 4°C for 4 h, and the immunoprecipitated beads were collected after washing with NT2 buffer 5 times. The beads were resuspended in NT2 buffer containing 0.3 mg/mL proteinase K and then incubated at 55°C for 30 min with shaking. After incubation, RNAiso Plus (#9109, Takara) was added to extract the immunoprecipitated RNA for PCR assays.

### Minigene Reporter Assay

To construct the *SRF* minigene plasmid, a genomic DNA fragment containing *SRF* exons 5-7 and a 100 bp flanking sequence was cloned into the pcDNA3.1(+) plasmid. According to the RBPmap website (http://rbpmap.technion.ac.il/), three *SRF* minigene mutant plasmids were constructed by mutating the sequence of exon 6 to determine the binding sites of the SRSF3 protein and *SRF* pre-mRNA. To investigate the effect of SRSF3 on the splicing of *SRF* minigene plasmids, minigene plasmids were cotransfected with *SRSF3* siRNA or overexpression plasmid into HCT-116 cells. The transcripts were amplified by RT–PCR with primers (FP1: TCATCCGTGCCCACAACTGT; FP2: GTTTCAGCAGTTCAGCTCCACC; RP1: CATTCATCTTGGTGCTGTGGG). The PCR products were then separated on agarose gels.

### Statistical Analysis

The relationship between SRF expression and clinicopathological features was analyzed by SPSS v26. The Pearson correlation coefficient was used to assess the correlation between SRSF3 expression and the other genes. For two-group analysis, a two-tailed Student’s *t* test was used to examine group differences. Significance threshold was p < 0.05. Data were analyzed and plotted using GraphPad Prism 8.0.1 (GraphPad Software Inc., USA).

## Results

### SRSF3 Mediated Gene Regulation in CRC

**Figure 1:**
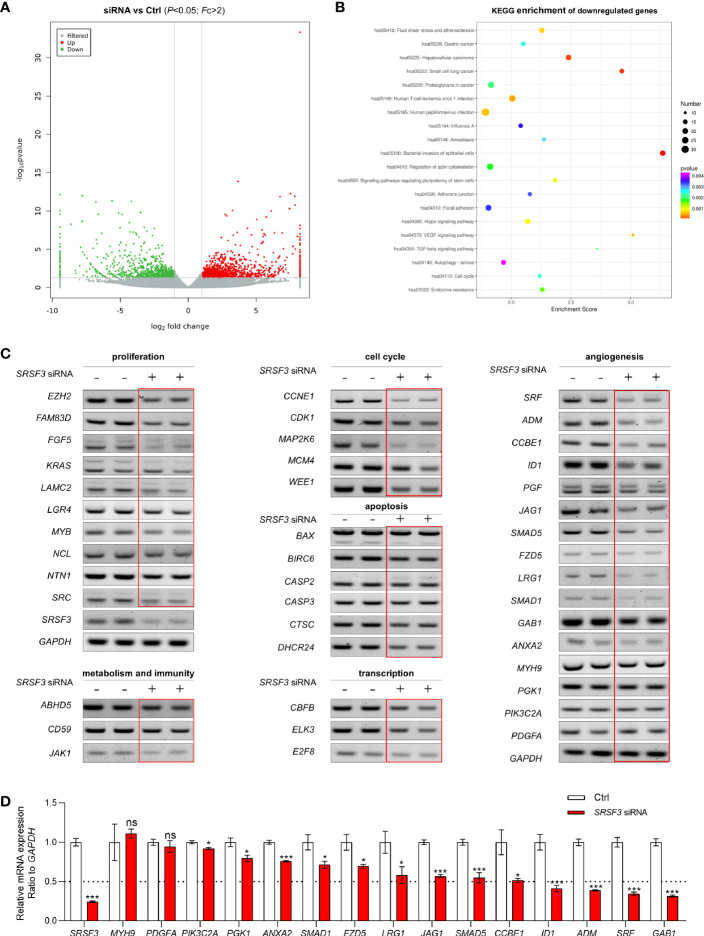
The expression and function of SRSF3-regulated genes in HCT-116 cells. **(A)** Clustered heatmap for differentially expressed genes regulated by SRSF3. The gene expression was measured by RNA-seq. **(B)** KEGG analysis of the SRSF3-downregulated genes. **(C)** RT–PCR assays for SRSF3-regulated genes involved in various biological processes (*n*=2). **(D)** qPCR assays for SRSF3-regulated genes related to angiogenesis (*n*=6). Data represent mean ± SD. Significance was assessed by two-sided *t* test. ***P < 0.001; *P < 0.05; ns, no significance.

SRSF3-regulated aberrant splicing is usually associated with numerous aspects of human cancers, such as the cell cycle, cytoskeleton, cell proliferation, apoptosis and other functions. To explore the role of SRSF3 in CRC, we first performed RNA-seq on the extracted RNA from HCT-116 cells transfected with *SRSF3* siRNA and siRNA control. The RNA-seq results showed that 1152 genes were upregulated and 870 genes were downregulated when SRSF3 was knocked down (**Figure 1A**). Then, we performed Kyoto Encyclopedia of Genes and Genomes (KEGG) pathway enrichment analysis on these downregulated genes (FC>2, P<0.05) and found that the downregulated genes were involved in the VEGF and TGF-β signaling pathways (**Figure 1B**). Therefore, the genes regulated by SRSF3 were involved in a variety of biological processes of cancer, suggesting that SRSF3 is expected to be a new therapeutic target for CRC.

Based on the RNA-seq results, we further screened and verified the expression of genes regulated by SRSF3 using RT–PCR assays. As shown in **Figure 1C**, the expression of many genes was decreased in the SRSF3-knockdown group, which indicated that SRSF3 regulated the expression of genes related to the cell cycle (*MAP2K6, WEE1, CDK1*), cell proliferation (*NCL, EZH2*), apoptosis (*CTSC, BIRC6*), immune function (*CD59, JAK1*), metabolism (*ABHD5*) and transcription factors (*ELK3, CBFB, E2F8*). Moreover, SRSF3 was positively correlated with genes related to angiogenesis, such as *ADM, SRF, ID1* and *GAB1*. These results were further verified by qPCR assay (**Figure 1D**).

### SRSF3 Regulated Angiogenesis-Related Genes

**Figure 2:**
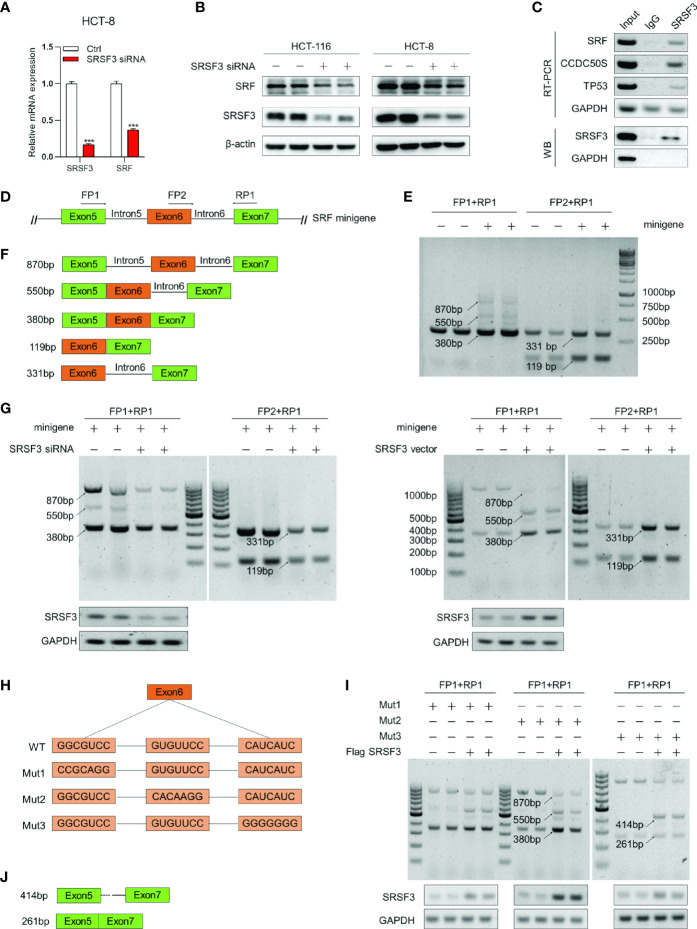
SRSF3-regulated splicing of *SRF*. **(A)** qPCR assays for analyzing the effects of SRSF3 knockdown on *SRF* mRNA expression in HCT-8 cells (*n*=6). **(B)** Western blotting to analyze the effects of SRSF3 knockdown on SRF protein expression in HCT-116 and HCT-8 cells (*n*=2). **(C)** RIP assays for analyzing the binding of SRSF3 protein with *SRF* pre-mRNA in HCT-116 cells. *GAPDH* was used as a negative control, while *TP53* and *CCDC50S* were used as positive controls (upper panels: RT–PCR for *SRF* mRNA, lower panels: immunoblotting for SRSF3 protein). **(D)** The schematic diagram of *SRF* mRNA and minigene. The *SRF* mRNA contains seven exons, and the predicted binding sites of SRSF3 were enriched on exons 5 to 7. The exons 5–7 were constructed into pcDNA3.1 vectors. Two forward primers (FP1 and FP2) and one reverse primer (RP1) were designed to amplify the transcripts of minigenes. **(E)** RT–PCR assays for investigating the transcripts of the *SRF* minigene in HCT-116 cells (*n*=2). **(F)** Schematic diagram and sizes of *SRF* minigene transcripts analyzed by DNA sequencing. **(G)** RT–PCR assays for investigating the effects of SRSF3 knockdown or overexpression on *SRF* minigene transcripts in HCT-116 cells (*n*=2). **(H)** Schematic diagram of three *SRF* minigene mutant plasmids. **(I)** RT–PCR assays for investigating the effects of SRSF3 overexpression on transcripts of *SRF* minigene mutant plasmids in HCT-116 cells (*n*=2). **(J)** Schematic diagram and sizes of *SRF* Mut3 plasmid transcripts analyzed by DNA sequencing. Data represent mean ± SD. Significance was assessed by two-sided *t* test. ***P < 0.001.

To further explore the target genes of SRSF3 regulating angiogenesis, the expression correlation between *SRSF3* and 16 angiogenesis-related genes based on the TCGA database was analyzed, as shown in **Supplementary Table 2**. Among these genes, SRF had a good correlation with SRSF3 expression. According to the results of qPCR and WB assays (**Figures 2A, B**), SRF mRNA and protein expression was significantly inhibited when SRSF3 was knocked down in HCT-116 and HCT-8 cells. Therefore, we concluded that SRSF3 had a positive regulatory effect on the expression of SRF mRNA and protein. SRF was chosen for subsequent experimental studies.

### SRSF3 Protein Directly Bound to *SRF* pre-mRNA

As an RNA binding protein, SRSF3 can bind to the binding sites on pre-mRNA and then participate in the splicing of target genes (30). Accordingly, we investigated whether SRSF3 could bind to *SRF* pre-mRNA and regulate *SRF* pre-mRNA splicing. We performed RIP assay to verify it. In this experiment, *CCDC50S* and tumor protein 53 (*TP53*) were chosen as positive controls, which have been identified as SRSF3 target genes (27, 31), while *GAPDH* was used as a negative control. As shown in **Figure 2C**, the SRSF3 protein in HCT-116 cells could be pulled down by the bead-antibody (SRSF3) complex. Meanwhile, the PCR results showed that endogenous *CCDC50S*, *TP53* and *SRF* mRNA could be detected in the SRSF3 protein-pulled down complex, while these could not be detected in the IgG control, as shown in **Figure 2C**. These results proved that the SRSF3 protein could directly bind to *SRF* mRNA.

### SRSF3 Regulated the Splicing of *SRF* pre-mRNA

To further explore the mechanism of SRSF3 splicing *SRF*, a minigene reporter assay was performed, and the design pattern of the *SRF* minigene is shown in **Figure 2D**. The *SRF* minigene plasmid and control plasmid were first transfected into HCT-116 cells. Then, the transcripts were analyzed by RT–PCR using specific primers. As shown in **Figure 2E**, amplicons of 870 bp, 550 bp and 380 bp were produced by FP1 and RP1, and amplicons of 331 bp and 119 bp were produced by FP2 and RP1. Compared with the control group, two amplicons (870 bp and 550 bp) were only detected in the *SRF* minigene group, and other amplicons (380 bp, 119 bp and 331 bp) were significantly enhanced. These results proved that the *SRF* minigene plasmid was successfully constructed. Furthermore, the above PCR products were sequenced, and the splicing patterns are shown in **Figure 2F**. To determine whether SRSF3 could affect the splicing of *SRF* pre-mRNA, the *SRF* minigene was cotransfected with *SRSF3* siRNA or expression plasmid into HCT-116 cells. The RT–PCR assay results showed that five transcripts (870 bp, 550 bp, 380 bp, 119 bp and 331 bp) were reduced when SRSF3 was knocked down, while four transcripts were enhanced except 870 bp when SRSF3 was overexpressed (**Figure 2G**). These results proved that SRSF3 could directly bind to *SRF* pre-mRNA and participate in the splicing of *SRF* pre-mRNA, thereby regulating SRF expression.

### SRSF3 Directly Bound to the “CAUC” Motif in Exon 6 of *SRF*

The RBPmap analysis showed that the potential binding sites of SRSF3 on *SRF* pre-mRNA were enriched in exon 6 of *SRF*. To determine the binding sites, we mutated three sequences in exon 6 separately and constructed three *SRF* minigene mutant plasmids, named Mut1, Mut2 and Mut3 (**Figure 2H**). Three mutant plasmids were cotransfected with the *SRSF3* expression plasmid into HCT-116 cells, and RT–PCR assays were performed to detect the changes in transcripts. As shown in **Figure 2I**, the group transfected with Mut1 and Mut2 plasmids had the same changes in transcripts as the WT group (the 870 bp transcript was reduced, while the other transcripts were enhanced). This result indicated that the mutant sequences of Mut1 and Mut2 were not the binding sites of SRSF3 and *SRF* pre-mRNA. Furthermore, transcripts of 414 bp and 261 bp were detected in the transfected Mut3 plasmid group compared with the WT group, but the 550 bp and 380 bp bands were almost undetectable. These results indicated that the mutated sequences of Mut3 changed the splicing mode of SRSF3 to *SRF* pre-mRNA, and the mutated sequence “CAUC” was the binding site of SRSF3 on *SRF* pre-mRNA. We also performed DNA sequencing on the 414 bp and 261 bp transcripts, and the splicing patterns are described in **Figure 2J**. The results showed that SRSF3 no longer binds to exon 6 when the binding sites in exon 6 were mutated, resulting in exon 6 skipping and partial intron retention.

### SRSF3 Was Highly Expressed in CRC

**Figure 3:**
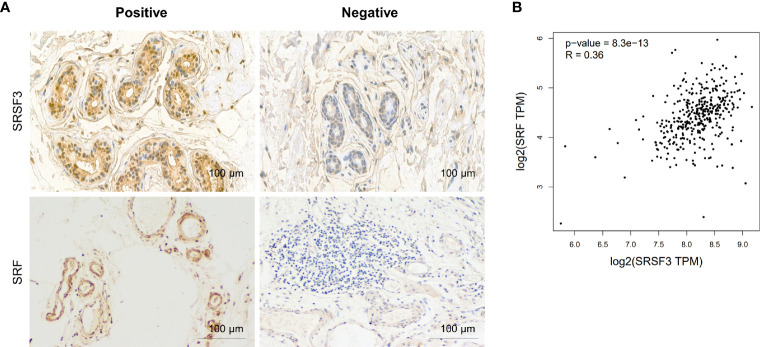
The expression of SRSF3 and SRF in CRC. **(A)** IHC staining of the SRSF3 and SRF proteins in endothelial cells around blood vessels (*n*=55). **(B)** The correlation between *SRSF3* and *SRF* mRNA expression in TCGA database.

Our previous study demonstrated that SRSF3 expression was enhanced in CRC tissues (32). Moreover, the IHC results showed that SRSF3 also had higher expression in perivascular tumor cells (**Figure 3A**). We also explored the expression of SRF in CRC. The results of the IHC assay showed that SRF was highly expressed in the endothelial cells around blood vessels (**Figure 3A**). Moreover, *SRSF3* mRNA expression was significantly correlated with *SRF* in the TCGA database (**Figure 3B**). In addition, the perivascular expression of SRF in CRC tissues was significantly positively correlated with SRSF3 expression and was associated with lymph node metastasis (**Table 1**). These results suggested that SRSF3 might be related to CRC angiogenesis and play an important role in the development of CRC.

**Table 1:** The relationship between SRF and clinical characteristics in CRC.

Features	Variables	SRF expression^a^		OR (95%CI)	*P* value^b^
		Low	High		
SRSF3	Low	10	10	**4.00 (1.20-13.36)**	**0.033**
	High	7	28		
Sex	Female	10	19	1.32 (0.43-4.04)	0.777
	Male	8	20		
Location	Colon	10	16	1.8 (0.58-5.55)	0.394
	Rectum	8	23		
size	<4 cm	6	10	1.45 (0.43-4.89)	0.545
	>4 cm	12	29		
Volume	<30 cm^3^	7	14	1.14 (0.36-3.59)	1
	>30 cm^3^	11	25		
T stage	T1/T2	8	10	2.32 (0.72-7.51)	0.221
	T3/T4	10	29		
N stage	N0	14	19	**3.68 (1.03-13.20)**	**0.048**
	N1/N2	4	20		
M stage	M0	12	35	0.69 (0.11-4.23)	0.649
	M1	2	4		
TNM stage	I/II	14	20	3.33 (0.93-11.91)	0.083
	III/IV	4	19		

a The expression levels of SRSF3 and SRF were classified as negative (score 0), low (score 1-2), and high (score 3-4).

^b^The P values less than 0.05 are in bold.

### SRSF3 Promoted the Angiogenesis of CRC

**Figure 4:**
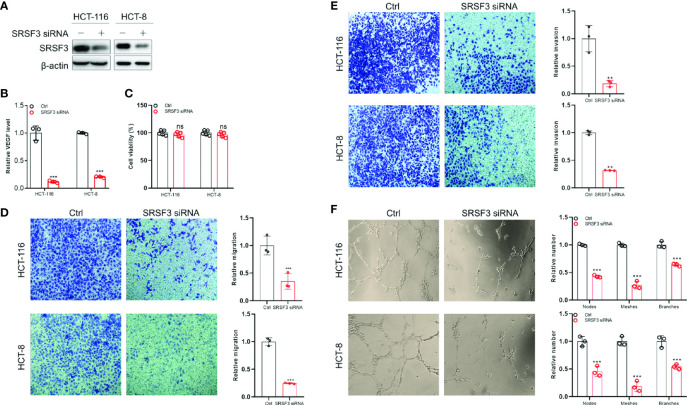
The SRSF3-promoted angiogenesis in CRC. **(A)** Western blotting for evaluating *SRSF3* siRNA in HCT-116 and HCT-8 cells. **(B)** ELISAs for detecting the effect of *SRSF3* siRNA on VEGF secretion in HCT-116 and HCT-8 cells (*n*=3). **(C)** MTT assays to investigate the effect of CM from SRSF3-silenced HCT-116 and HCT-8 cells on the proliferation of HUVECs (*n*=6). **(D)** Transwell assays for investigating the effect of CM from SRSF3-silenced HCT-116 and HCT-8 cells on the migration of HUVECs (*n*=3). **(E)** Transwell assays for investigating the effect of CM from SRSF3-silenced HCT-116 and HCT-8 cells on the invasion of HUVECs (*n*=3). **(F)** The effects of CM from SRSF3-silenced HCT-116 and HCT-8 cells on the tube formation of HUVECs (*n*=3). Data represent mean ± SD. Significance was assessed by two-sided *t* test. ***P < 0.001; **P < 0.01; ns, no significance.

Since SRSF3 regulated angiogenesis-related genes, we wondered whether SRSF3 would affect VEGF secretion and angiogenesis in CRC. Therefore, we first performed ELISA to measure VEGF protein expression in CM from SRSF3-knockdown HCT-116 and HCT-8 cells (**Figure 4A**). We found that VEGF protein expression in CM from SRSF3-knockdown HCT-116 or HCT-8 cells was significantly decreased compared with that in CM from scrambled control cells (**Figure 4B**). Next, we used CM from SRSF3-knockdown HCT-116 or HCT-8 cells to explore the effects of SRSF3 on the proliferation, migration, invasion and tube formation of HUVECs. We found that CM had no effect on the proliferation of HUVECs (**Figure 4C**), but significantly reduced the number of HUVECs that migrated or invaded the Transwell chambers compared with the scrambled control (**Figures 4D, E**). Meanwhile, the results of the tube formation assay shown in **Figure 4F** demonstrated that the number of nodes, meshes and branches in SRSF3-knockdown CM was significantly lower than that in the control group. The above results indicated that CM from SRSF3-knockdown HCT-116 or HCT-8 cells significantly inhibited the migration, invasion and tube formation of HUVECs, which verified the important role of SRSF3 in CRC angiogenesis.

### SRSF3 Promoted the Angiogenesis of CRC by Regulating SRF

**Figure 5:**
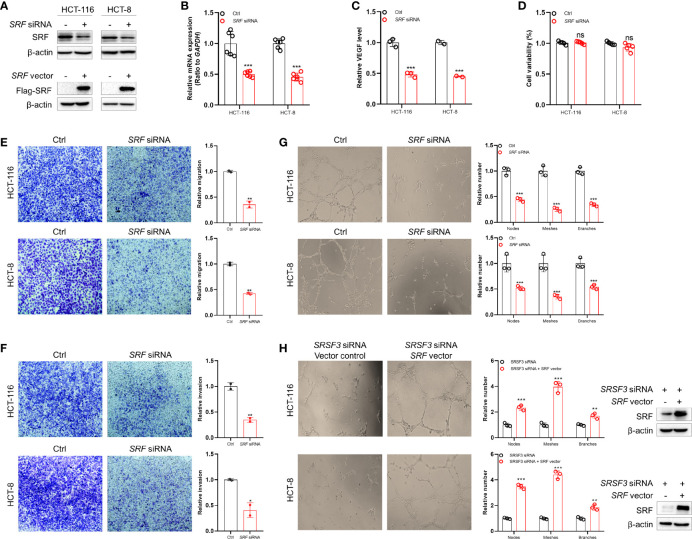
The SRF-promoted angiogenesis in CRC. **(A)** Western blotting assays for verifying *SRF* siRNA and expression vector in HCT-116 and HCT-8 cells. **(B)** qPCR assays for verifying *SRF* siRNA in HCT-116 and HCT-8 cells (*n*=6). **(C)** ELISAs to investigate the effects of SRF silencing on VEGF secretion in HCT-116 and HCT-8 cells (*n*=3). **(D)** MTT assays for investigating the effects of CM from SRF-knockdown HCT-116 and HCT-8 cells on the proliferation of HUVECs (*n*=6). **(E)** Transwell assays to investigate the effects of CM on the migration of HUVECs (*n*=2). **(F)** Transwell assays to investigate the effects of CM on the invasion of HUVECs (*n*=2). **(G)** The effects of CM on the tube formation of HUVECs (*n*=3). **(H)** The effects of CM from HCT-116 and HCT-8 cells transfected with *SRSF3* siRNA and *SRF* expression plasmid on the tube formation of HUVECs (*n*=3). Data represent mean ± SD. Significance was assessed by two-sided *t* test. ***P < 0.001; **P < 0.01; ns, no significance.

To investigate the role of SRF in CRC angiogenesis, HCT-116 and HCT-8 cells were first transfected with *SRF* siRNA or expression plasmid, and then CM was collected. The results of western blot and qPCR assays demonstrated that *SRF* siRNA and expression plasmids regulated SRF expression at the mRNA and protein levels (**Figures 5A, B**). As shown in **Figure 5C**, the ELISA results showed that the secretion of VEGF was significantly decreased in SRF-knockdown CM. We further investigated the effect of SRF-knockdown CM on the proliferation, migration, invasion and tube formation of HUVECs. Consistent with the SRSF3 knockdown results, SRF-knockdown CM had no effect on the proliferation of HUVECs (**Figure 5D**). However, the number of HUVECs crossing the Transwell chambers was significantly lower than that crossing the scrambled control, regardless of the presence of Matrigel in the chambers (**Figures 5E, F**). Moreover, SRF-knockdown CM induced HUVECs to develop fewer tubes than the scrambled control (**Figure 5G**). These results showed that SRF-knockdown CM could significantly inhibit the migration, invasion and tube formation of HUVECs, suggesting that SRF also played an important role in CRC angiogenesis.

To explore whether SRSF3 promoted the angiogenesis of CRC by regulating SRF, we performed rescue experiments by cotransfecting *SRSF3* siRNA with an *SRF* expression plasmid into HCT-116 and HCT-8 cells. As shown in **Figure 5H**, the results showed that the overexpression of SRF diminished the inhibitory effect of SRSF3 knockdown on HUVEC tube formation, indicating that SRSF3 promoted the angiogenesis of CRC by regulating SRF.

## Discussion

The high morbidity and mortality of CRC remain a worldwide challenge. Abnormal angiogenesis is one of the common clinical traits of CRC. In this study, we demonstrated that SRSF3 was highly expressed in CRC tissues and tumor cells around blood vessels and verified that SRSF3 played important roles in the angiogenesis of CRC. Moreover, our data showed that SRSF3 positively regulated SRF expression and consequently promoted CRC angiogenesis by driving the migration, invasion and tube formation of HUVECs.

Alternative splicing is a common process leading to transcript variation and proteome diversity (17), and aberrant splicing is usually the cause of cancer occurrence and development. It has been reported that aberrant splicing leads to an increase in proangiogenic isoforms of *VEGFA* and prompts tumor angiogenesis (33). For example, aberrant splicing-mediated upregulation of BCL2-like 1 (*BCL2L1*) antiapoptotic isoform enhances the antiapoptotic ability of cancer (34). Aberrant splicing is commonly caused by mutations or abnormal expression of splicing factors. SRSF3 is a member of the SR protein family with the highest expression in CRC (35), which regulates the alternative splicing of multiple genes and participates in numerous steps of RNA biological metabolism. Previous studies have shown that SRSF3 expression could be used as a biomarker for cancer diagnosis and prognosis (29, 36). In this study, we first performed IHC staining on 55 CRC tissues, and the results showed that SRSF3 had a higher expression in CRC tissues, which was consistent with other studies (35, 37, 38). In addition, SRSF3 was also highly expressed around tumor blood vessels. These results suggested that SRSF3 could have a positive effect on the angiogenesis of CRC. Then, we performed RNA-seq on HCT-116 cells transfected with *SRSF3* siRNA or negative control. The results showed that genes regulated by SRSF3 are involved in a variety of biological processes, such as the cell cycle, proliferation, migration, invasion, cell metabolism and immune response. Moreover, the results of RT–PCR and qPCR assays showed that SRSF3 regulated the expression of genes related to angiogenesis, including *SRF*. We utilized RIP assays and minigene reporter assays to prove that SRSF3 participated in the splicing of *SRF* pre-mRNA by binding to the “CAUC” motif in exon 6, thereby regulating *SRF* expression.

SRF, a member of the MADS box superfamily of transcription factors, mediates the transcription of genes related to cell growth, migration, cytoskeleton, and energy metabolism and regulates the expression of cell adhesion factors (39, 40). SRF is highly expressed in gastrointestinal cancers, such as hepatocellular carcinoma, CRC and esophageal cancer. It also regulates the Wnt/β-catenin pathway, promotes the expression of MMP2, MMP9 and E-cadherin/β-catenin, and enhances the proliferation, invasion and angiogenesis of tumor cells, thereby promoting tumor metastasis (41). VEGF is one of the major factors that initiates and regulates angiogenesis (42). Several studies have found that SRF is a downstream mediator of VEGF signal transduction in endothelial cells and is also a key condition for VEGF-induced angiogenesis (43, 44). VEGF can induce SRF expression and nuclear translocation through the MEK-ERK and Rho GTPase signaling pathways and increase the binding activity of SRF to DNA in endothelial cells (45).

Our study demonstrated that SRF played a proangiogenic role in CRC. We performed IHC staining on 55 CRC tissue samples for SRF, and the results showed that SRF was highly expressed not only in CRC tissues but also around tumor blood vessels. Meanwhile, SRF could act as an upstream regulator to affect the expression of VEGF. The ELISA results showed that the expression of VEGF was downregulated in the CM from SRF-knockdown HCT-116 and HCT-8 cells, indicating that there was a mutual regulatory relationship between SRF and VEGF. Moreover, CM from SRF-knockdown HCT-116 and HCT-8 cells significantly inhibited the migration, invasion and tube formation of HUVECs. We also found that the overexpression of SRF reversed the inhibitory effect of SRSF3 knockdown on HUVEC tube formation. Our study suggested that SRF played an important role in the angiogenesis and development of CRC.

In summary, we confirmed that SRSF3 promoted the angiogenesis of CRC by regulating SRF through a series of *in vitro* experiments. SRSF3 could directly bind to *SRF* pre-mRNA and participate in the splicing of *SRF* pre-mRNA, thereby positively regulating SRF expression. In short, our study provides a theoretical basis for SRSF3 as a therapeutic target for CRC and provides a new direction for the treatment of CRC.

## Data Availability Statement

The datasets presented in this study can be found in online repositories. The names of the repository/repositories and accession number(s) can be found in the article/**Supplementary Material**.

## Ethics Statement

The studies involving human participants were reviewed and approved by The Ethics Committee of Soochow University. The patients/participants provided their written informed consent to participate in this study.

## Author Contributions

WW, JS, and HYZ conceived the idea and directed the study. YC, YZ, MY, HYZ, JS, and WW designed the experiments and wrote the manuscript. YC, YZ, MY, JY, and FM performed experiments. YC, YZ, and WW analyzed the data. YC, MY, YZ, HYZ, and WW generated figures. WW, JS, and HYZ critically revised the manuscript for important intellectual content. All authors contributed to the article and approved the submitted version.

## Funding

This research was supported by the National Natural Science Foundation of China (No. 81773044), Science and Technology Special Project of Clinical Medicine in Jiangsu Province (BL2014046), Social Development Project of Jiangsu Province (BE2019657), Qinglan Project of Jiangsu Province, and the Priority Academic Program Development of Jiangsu Higher Education Institutions (PAPD).

## Conflict of Interest

The authors declare that the research was conducted in the absence of any commercial or financial relationships that could be construed as a potential conflict of interest.

## Publisher’s Note

All claims expressed in this article are solely those of the authors and do not necessarily represent those of their affiliated organizations, or those of the publisher, the editors and the reviewers. Any product that may be evaluated in this article, or claim that may be made by its manufacturer, is not guaranteed or endorsed by the publisher.
