# Supplementary material for: SRSF3 Promotes Angiogenesis in Colorectal Cancer by Splicing SRF
Source: Front Oncol. 2022 Feb 7;12:810610. doi: 10.3389/fonc.2022.810610 (PMC8859257; doi:10.3389/fonc.2022.810610)
Supplement: Supplementary file 1 [file DataSheet_1.pdf]

Supplementary Materials

Table S1. Primers for RT-PCR and qPCR.

Gene	Forward primer (5’→3’)	Reverse primer (5’→3’)
ABHD5	GGACGAAGTAGTAGACCCAGGTT	GGTACTTCAGCGAGTAAGCAGCA
ADM	GCGCAAGCCTCACTATTACTTG	ACACCTCGCTGAGACATTCAC
ANXA2	GAGACAGGATATTGCCTTCGCCTA	GCGTCATACTGAGCAGGTGTCTT
BAX	CAGCTCTGAGCAGATCATGAAGAC	CAAAGTAGAAAAGGGCGACAACCC
BIRC6	GGCTACCCTTGCTTCCTCTTCT	GAATAGGAGCAACTAGGTCGGC
CASP2	ACTGCCCAAGCCTACAGAACAA	TCTTTACCGGCATCACTCTCCT
CASP3	CAGAGGGGATCGTTGTAGAAGTC	ATGAACCAGGAGCCATCCTTTG
CBFB	GGCCACAGGAACCAATCTGT	GGCTCGCTCCTCATCAAACT
CCBE1	TACTCGGGAAGCTGAGACACAAG	CTCAGAAGGTGAGAAAAGGCACCA
CCDC50	GCTGGCTATTGAGGCAGAG	TGGCTTCATTCCTCCATCTT
CCNE1	CCAAACTCAACGTGCAAGCC	CTCCTGAACAAGCTCCATCTGTC
CD59	GCTGTCTTCTGCCATTCAGGTC	GGAGTCACCAGCAGAAGAACTG
CDK1	CTACAGGTCAAGTGGTAGCCAT	TGTACTGACCAGGAGGGATAGA
CTSC	CCTACACAGGCACTGATTCTCCAT	TAGCCCACAAGCAGAACAGCAT
DHCR24	ATGGAAGGAGCAGGGTAGCA	AAGGGCTCCACACGGACAAT
ELK3	AAGACCAAGTCTCCATCTCTTCCC	GAAATGTATGCTGGAGAGCAGTGG
EZH2	CCACAGTGTTACCAGCATTTGGAG	CTGCTTCCCTATCACTGTCTGTATCC
E2F8	GTGTCAACGCCTCAGATAGTAAGC	TGGTTTTCGGCCTCTTTCCTC
FGF5	TATGTCTTCCTCTTCTGCCTCCTC	CTCCTCGTATTCCTACAATCCCCT
FZD5	GCTCTGCTGCCTTCAGTCATT	GTGCTTCTGAAAGTTGGTCTGG
FAM83D	ACACAAACCAGCATCACCACAG	CCCTTCTGTAGATTGAGTTCCTCG
GAB1	GGGACAGACATCAAAGCTAGACAC	ATTACTACGTGAAGGCGACATCC
ID1	GGTAAACGTGCTGCTCTACGAC	CCTGATGTAGTCGATGACGTGC
JAG1	TCAGTTCGAGTTGGAGATCCTGTC	AAACTGAAAGGCAGCACGATGC
JAK1	TCTCCTTGATGCCAGCTCACT	AGCCATCCCTAGACACTCGTT
KRAS	CAGGCTCAGGACTTAGCAAGAAG	TAACAGTCTGCATGGAGCAGG

Gene	Forward primer (5’→3’)	Reverse primer (5’→3’)
LAMC2	GAAGAGTGAGATGAGGGAAGTGGA	GCCGTCTAATGTGTTGAGTGTGTC
LGR4	GGCTCAGCGCCTTCACCCAA	GCACTCAGCCCTCGAATGGC
LRG1	ACCTCTATCGTTGGCTTCAGG	GTTGCAGTGTTCTACCAGACC
MAP2K6	CCAGTCTGTTTTGCAAGGTGTG	TGTGGAACTGGTCTGAGGTTGT
MCM4	CAGAGACGTAGAGGCGAGGATT	GTGCCGTATGTCAGTGGTGAAC
MYB	CCAGGGCACCATTCTGGATAA	GGTGTAGGAGTTCTTGGAGAGC
MYH9	AAGAAGCTGGTATGGGTGCCT	GTAGTAACGCTCCTTGAGGTTGTG
NCL	AAGTGGAAGGCACAGAACCGA	GTGTTCTCGCATCTCGCTCTTTC
NTN1	CAAAACCTGCAACCAAACCACC	TAGGAATCGCAGTCTTCAGGCT
PDGFA	GAGGAAGAGAAGCATCGAGGAAG	GCTTCTTCCTGACGTATTCCACC
PGF	ACCATGCAGCTCCTAAAGATCC	AGGCATTCAGCAGGGAAACAG
PIK3C2A	ATTACCTGGGCCTTCCACTTATGC	AAAGGGTGTGGCAGGTGTCAAA
SMAD1	TTCTTTCCAGCAACCCAACAGC	CGCCTGAACATCTCCTCTGTT
SMAD5	CCCAGCCTATGGATACAAGCAA	GCATACACCTCTCCACCAACATAG
SRC	CTGTTCGGAGGCTTCAACTCCT	CACCAGTCTCCCTCTGTGTTGT
SRF	CGCGTGAAGATCAAGATGGAGT	TTCTCTGGTCTGTTGTGGGGT
SRF b	AAGAGGGCCCTTGCTGAGTGAA	CCTGGTAGGTGAGATCTGTCTC
SRF FP1	TCATCCGTGCCCACAACTGT	
SRF FP2	GTTTCAGCAGTTCAGCTCCACC	
SRF RP1	CATTCACTCTTGGTGCTGTGGG	
SRSF3	GGAAACAATGGCAACAAGACGG	TTCTAGCAACCCACACACTTCG
TP53	CTCTGACTGTACCACCATCCACTA	GAGTTCCAAGGCCTCATTCAGCTC
WEE1	CAGTGTCGTCGTAGAAAGAGAACG	ATCCATCCAGCCTCTTCACACA
GAPDH	TGCACCACCAACTGCTTAGC	GGCATGGACTGTGGTCATGAG
β-actin	AGAAAATCTGGCACCACACC	AGAGGCGTACAGGGATAGCA

Table S2. The correlations between *SRSF3* expression and angiogenesis-related genes

Gene	*SRSF3*	
	**R**	**P**
PGK1	0.49	0
PIK3C2A	0.48	0
SMAD1	0.43	0
SMAD5	0.37	1.1×10^{-13}
SRF	0.36	8.3×10^{-13}
GAB1	0.28	6.5×10^{-8}
FZD5	0.27	1.4×10^{-7}
JAG1	0.21	3.8×10^{-5}
MYH9	0.19	1.9×10^{-4}
ANXA2	0.15	3.1×10^{-3}
ADM	0.12	2.6×10^{-2}
ID1	0.08	0.13
PGF	-0.03	0.56
CCBE1	-0.05	0.39
LRG1	-0.08	0.11
PDGFA	-0.09	0.1

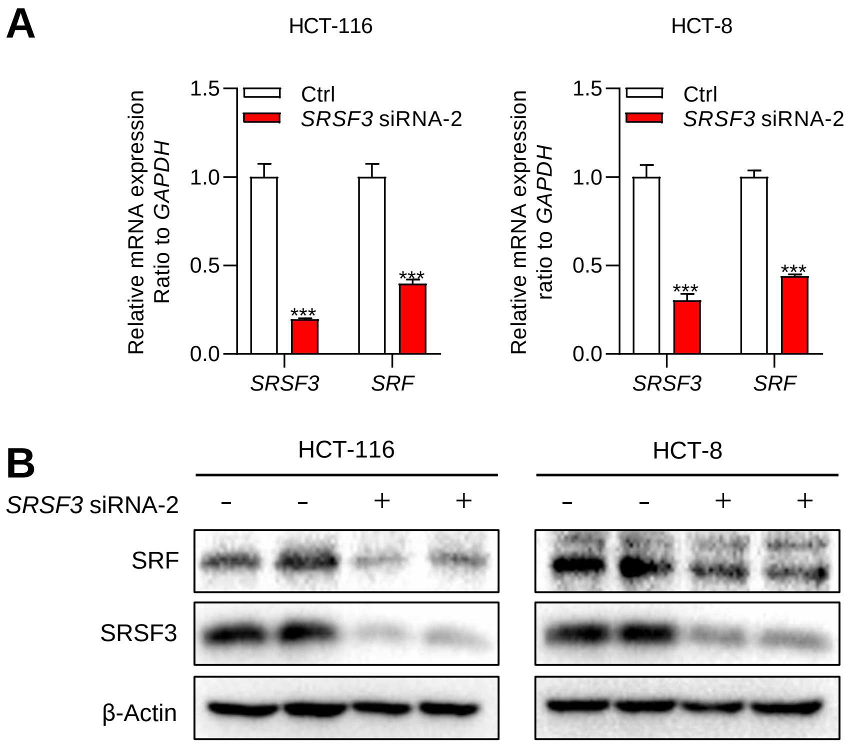

Figure S1. *SRSF3*-regulated splicing of *SRF*. (**A**) qPCR assays for analyzing the effects of *SRSF3* siRNA-2 on *SRF* mRNA expression in HCT-116 and HCT-8 cells (*n*=6). (**B**) Western blotting for analyzing the effects of *SRSF3* siRNA-2 on SRF protein expression in HCT-116 and HCT-8 cells (*n*=2).
